# ‘Who Listens to the Listener, Who Cares for the Carer?’ A Cross‐Sectional Study of Social Connectedness and Sleep Experiences of Young Siblings of Neurodivergent People

**DOI:** 10.1111/cch.70014

**Published:** 2024-12-04

**Authors:** G. Pavlopoulou, E. Sim, S. Peter, M. Gardani, V. Beevers, C. Kassa, V. Sideropoulos

**Affiliations:** ^1^ Group for Research in Relationships in Neurodiversity (GRRAND), Department of Clinical, Education and Health Psychology, Division of Psychology & Language Sciences, Faculty of Brain Sciences University College London London UK; ^2^ Anna Freud National Centre for Children and Families London UK; ^3^ Department of Psychology & Human Development, IOE, UCL's Faculty of Education and Society University College London London UK; ^4^ School of Health in Social Science University of Edinburgh Edinburgh UK; ^5^ CEO Sleep Charity; ^6^ CEO Sibs Charity

**Keywords:** disability, loneliness, mental health, siblings, sleep

## Abstract

**Background:**

A growing body of research postlockdown has established that loneliness and sleep problems are prominent in the life of all people and in particular in neurodivergent people and their parents/carers. The present study explores the experience of loneliness and sleep in siblings of neurodivergent young people.

**Methods:**

Thirty‐eight (*n* = 38) young siblings (*M*_age_ = 16.41, 68.4% female) completed an online survey on sleep, loneliness and daytime functioning, answering a set of qualitative questions.

**Results:**

Thematic analysis revealed that their sleep was affected by personal anxieties and neurodivergent siblings' parasomnias. Definition of loneliness included perceived lack of understanding and empathy in wider society, assuming a lonesome responsibility, growing up faster than peers and an emptiness within and without. Siblings provided brief contributions on how schools and the wider society can help them. Limitations include small sample size and an uneven representation of gender and disability groups in the sample. Recommendations for school and societal support are also discussed.

**Conclusion:**

This preliminary exploration helped define their caring responsibilities, social connectedness and sleep needs. Our findings call for a holistic and personalised approach to healthcare, including social and psychological support, for the whole family including neurodivergent and neurotypical siblings.

**Key Messages**

1. The prevalence of loneliness is on the rise.
2. Existing literature is limited on understanding the experience of loneliness and sleep in siblings of neurodivergent young people.
3. The present study explores loneliness and sleep experiences of siblings of neurodivergent young people.
4. Personalised and holistic approaches to social and psychological support could benefit all family members including siblings.

## 1 Introduction

There are more than 13.3 million people in the United Kingdom living with a disability and/or chronic illness, of which 660 000 are children below the age of 16 (Family Resources Survey, 2019). Amongst persons with disabilities and/or chronic illnesses, 99.1% of them are living at home and are supported by their families (Family Resources Survey, 2019). Of the informal care provided to disabled and/or chronically ill individuals, 10% are sibling young carers aged below the age of 25, with more females providing care as compared with males. Based on a new mixed methods systematic review (Wolff et al. 2022), it is reported that siblings of neurodivergent people are experiencing anxiety, depression and loneliness; however, there are considerable gaps in the factors associated with those mental health outcomes in siblings. In this paper, we echo Pavlopoulou et al. (2022) that there is a clear need to look in greater depth of social determinants in siblings' mental health by involving siblings across all stages of research.

Sibling relationships are often considered to be some of the longest and most influential relationships that individuals may have in their lives (White and Hughes 2018; Whiteman, McHale, and Soli 2011), and relationships during childhood are key predictors for depression 30 years later in life (Waldinger, Vaillant, and John Orav 2007). On the other hand, sibling relationships can be a key learning facilitator for various aspects. For example, neurotypical siblings who have a disabled or neurodivergent sibling often develop a better understanding and acceptance of neurodivergences (Meltzer 2018) as well as empathy (Shivers 2019). As such, the experiences of siblings are an important consideration for both individuals' and families' mental health. There is a growing body of research on the impact of siblings on one another's well‐being, with studies finding that siblings can play a significant role in shaping each other's emotional, social and physical development (Dunn 2011; Green 2013; White and Hughes 2018). It is estimated that up to 50% of young carers (under 18 years of age) worldwide provide care for a sibling, although this figure may vary by culture. However, it is unclear to what extent these siblings may provide a day‐and‐night caring and how this role may affect them. Siblings of individuals with disabilities such as autism, down syndrome or chronic illness, for example, epilepsy or cystic fibrosis, may face isolation and bullying as they navigate life with their neurodivergent sibling demands and barriers of living by neurotypical norms and rules (Mokoena and Kern 2022).

Most of the previous literature on siblings of children with a disability, health issues or both has focused on their functioning or adjustment compared with siblings of children who do not have an autistic brother or sister. For example, a meta‐analysis of 69 articles, (Shivers, Jackson, and McGregor 2019) found that nondisabled siblings of autistic children have significantly worse outcomes across all areas of social, emotional, behavioural and psychological functioning than comparison groups. Siblings are rarely asked to self‐report aspects of their lives and how they make meaning of their roles at home, school and their local communities. Several qualitative studies have explored family detachment or sleep problems in isolation usually by asking parents to report on their experiences and commonly of their children. Such studies show that family members may have limited opportunities to engage in social activities because of caring responsibilities or inaccessible community and leisure opportunities (Carr 2013; Heiman 2002). During the COVID‐19 pandemic, Kassa and Pavlopoulou (2021) surveyed 864 parents who reported that one in three siblings were feeling lonely and had no access to respite time with peers and extended family members. Similarly, Sideropoulos, Dukes, et al. (2022) and Sideropoulos, Kye, et al. (2022) provide further evidence for the deterioration of both neurodivergent and neurotypical's mental health during the COVID‐19 pandemic not only in the United Kingdom but also across the world (Sideropoulos et al. 2023). Until now, we have limited reports focusing on siblings' sense of connectedness and the potential impact of day and night‐time caring responsibilities. Of concern to the mental health and adjustment in neurotypical siblings are feelings of loneliness. Similarly, neurotypical siblings face a myriad of factors that may predispose them to loneliness. These include lack of empathy from the public (Stalker and Connors 2004), additional caring roles with little respite and leisure time (Welch et al. 2012), societal stigmatisation (Mazumder and Thompson‐Hodgetts 2019) and proneness to school bullying (Evans, Jones, and Mansell 2001). Loneliness is defined as the undesirable feeling when one's social relationships are perceived to be poor, leading to a desire to belong and feel socially connected (Perlman and Peplau 1982). It is well known that loneliness is often a contributor to an individual's subjective well‐being (Asher and Paquette 2003) and is a predictor of poor mental health across ages, cultures, gender identities and socio‐economic backgrounds (Deacon et al. 2018; Savikko et al. 2005).

Sleep problems affect up to 86% of children with disabilities and/or chronic illnesses. The most common sleep problems are sleep onset delay, sleep maintenance and parasomnias. Difficulties relating to sleep in childhood populations may affect the quality of life for the entire family (Wiggs and France 2000). Many researchers have found that loneliness is a strong predictor of poor sleep. Systematic review and meta‐analytic studies have shown that loneliness is related to sleep issues and health conditions (Griffin et al. 2020; Wang et al. 2018; Deckx, van den Akker, and Buntinx 2014). Nonetheless, there is very limited research on siblings' sleep and social connectedness experiences and needs and their potential impact on siblings.

Feeling lonely can also lead to feelings of sadness, frustration and stress, which can ultimately affect their sleep quality. Research has also found that siblings of disabled individuals may experience disrupted sleep patterns and are at higher risk of health problems (Cooke et al. 2024). This can be due to a variety of factors, such as caring for their sibling during the night, worry and anxiety about their sibling's condition and difficulty managing their own emotions. To address the impact of loneliness and sleep experiences on siblings of disabled or chronically ill individuals, it is important to first understand loneliness in children and young people as the research in siblings of chronically ill and disabled sisters and brothers is limited.

### 1.1 Current Study

This exploratory study's aim was to uncover the experiences, needs and perceived links if any from the perspective of siblings who grow up and care for a neurodivergent brother or sister. Importantly, we invited siblings who spend a considerable amount of time in day and night time caring activities with their siblings to examine how they perceive the day‐to‐day experiences of loneliness and sleep habits in relation to their family life using the qualitative survey.

## 2 Method

### 2.1 Community Participation

Our team of academics partnered with two charities that have recently joined forces to respond to families' and siblings' anecdotal evidence regarding siblings' lives. The charity staff from both organisations are also experts by experience, with professional expertise in supporting neurotypical and neurodivergent siblings. In line with participatory approaches described by Fletcher‐Watson et al. (2021), the first author invited three adult siblings as experts by experience and two sleep trainers who are also experts by experience to join as coresearchers. The first author had participated in discussions with the two charities on how to best support siblings and explicitly invited them to join forces with the academic team. During the initial stage of consultations, we held one‐on‐one meetings and exchanged emails to elicit targeted, expert advice on the project at key intervals. We arranged specific areas of responsibility, such as co‐creating and piloting survey items. We soon became a partnership to discuss and validate the research aims, methods and results of the study and to obtain recommendations for future research. During the partnership stage, all partners transitioned from advisors to coresearchers and shared authorship. Our expert coresearchers from Sibs charity and Sleep charity discussed their observations with the authors and explained how the results of this study have been consistent with their observations as well as commented on novel results and suggested ways of disseminating all via their national channels.

### 2.2 Participants

The original sample consisted of 52 participants; 14 were removed because they did not fit the inclusion criteria for this study: (a) living in an English‐speaking country; (b) aged between 8 and 25 years old; and (c) living in the same household to their neurodivergent brother or sister or in close distance that allows them to engage in caring responsibilities. The final sample included 38 participants (*M*_age_ = 18.42, *SD*_age_ = 5.43) who identified as neurotypical and who had a neurodivergent sibling. The study was advertised on social media and to charities like Sibs UK, The Sleep Charity, the National Autistic Society and the London Autism Group Charity, which all agreed to advertise it on their social media platforms. Characteristics of the participants including family backgrounds and employment can be found in Table 1.

**TABLE 1:** Demographic information of participants (neurotypical siblings) including employment, education and type of family.

Demographics	*n*	%
Gender		
Male	12	31.6
Female	26	68.4
Country of residence		
England	25	65.8
Northern Ireland	2	5.4
Wales	2	5.4
Other (e.g., Singapore and Canada)	9	23.7
Type of employment		
Working	6	15.8
Studying	26	68.4
Working and studying	4	10.5
Other	2	5.3
Level of education		
Primary	11	28.9
Secondary	8	21.1
A levels	5	13.2
Bachelors	6	15.8
Masters	5	13.2
Other	3	7.9

The neurotypical sibling provided self‐reported details of their sibling's primary and additional diagnoses. The age range for participant's neurodivergent siblings was between 4 and 31 years (*M* = 16.41, *SD* = 5.91), and all lived with their parents and/or their neurotypical siblings. Most neurotypical siblings reported that their neurodivergent sibling presented with more than one condition. Autism was the second most reported condition followed by Down syndrome; all other conditions were characterised by learning disabilities with co‐occurring medical conditions such as epilepsy and/or myoskeletical issues and were grouped together. Characteristics of the neurotypical participant's disabled siblings can be found in Table 2.

**TABLE 2:** Demographic information of participant's disabled siblings.

Demographics	*n*	%
Gender		
Male	26	68.4
Female	9	23.7
Other	1	2.6
Missing	2	5.3
Diagnosis		
Autism	9	23.7
Down syndrome	4	10.5
Learning disability and co‐occurring autism/ADHD and mental health struggles	18	47.4
Learning disabilities (e.g., cerebral palsy or ADHD)	6	15.8
Missing	1	2.6

### 2.3 Measures

There are 21 open‐ended questions relating to their activities and routines from 4 p.m. until they fall asleep, the type of involvement they might have in the routines of their siblings, any night‐time caring activities, description of key sleep problems of their disabled/chronically ill brothers or sisters and strategies they find useful when they want to offer help as well as their own difficulties and strategies, as well as social experiences and needs. Examples of the qualitative questionnaire are presented in Table S1.

### 2.4 Procedure

Thirty‐eight participants completed an online survey hosted in Qualtrics. The survey was divided into three sections: (a) a demographics section where participants completed questions about their personal background and their brother/sister and (b) a set of open‐ended questions.

### 2.5 Ethics

The study obtained ethical approval from the Dissertation Ethics Committee in the Department of Psychology & Human Development at IOE, UCL's Faculty of Education and Society. All respondents provided online consent to participate in the study.

### 2.6 Qualitative Analysis

A set of descriptive statistics were computed to investigate the levels of sleep, sleepiness, loneliness and psychological adjustment of the neurotypical and neurodiverse populations. Data obtained from open‐ended questions were analysed using thematic analysis. Typically, the responses to the open‐ended questions were short; the range was from a few words up to two sentences. Thematic analysis is a flexible method that can be used to identify patterns within data without a pre‐existing theoretical framework (Braun and Clarke 2006; Clarke, Braun, and Studies 2013). We analysed the data using reflexive thematic analysis (Braun and Clarke 2006, 2021) with a blended inductive/deductive approach. That means that few codes were set up as a starting point deductively (for instance, ‘sleep habits’ or ‘experiences of loneliness’), but then the research team came up inductively with new codes and iterated on pre‐existing codes as we sifted through our data. We used reflexive thematic analysis to facilitate a flexible yet detailed interpretation of the dataset and a blended approach to facilitate consideration of the data in relation to Frost's model (2021), alongside new interpretations that arose during the coding process. We drew upon a critical realist framework (Botha 2021; Kourti 2021) in our interpretations of the data. We remained mindful that our positionality may influence our own interpretations because of personal, academic and professional experiences. Initially, ES read and re‐read each open‐ended answer line‐by‐line and attached some pre‐existing codes. ES and GP systematically identified interesting and relevant information embedded within the data and assigned new codes (for instance, ‘feeling misunderstood’ and ‘feeling lonely’). Two different lists were created, one containing sleep‐coded answers and one containing loneliness‐related answers. The themes emerging from the data relevant to the subject under investigation were coded. Preliminary notes were made in each document during each reading, and a theme table was constructed for each document, identifying and describing relevant sections of data. Themes were subsequently grouped, and a coding framework was devised for the complete dataset (Braun and Clarke 2006). Emerging themes were then discussed and compared by the authors. This process ensures greater exploration of the data and increases both the validity and reliability of resulting themes (Denzin and Lincoln 2008).

## 3 Results

### 3.1 Learning From Siblings About Sleep, Loneliness and Daytime Function

#### 3.1.1 What Did We Learn From Siblings About Sleep?

Siblings provided short open answers to survey questions that were codeveloped by authors VK and CK who are experts by experience and work closely with our community partners. The findings were grouped into (1) nondisabled siblings' accounts on common sleep issues of neurodivergent brother or sister, their roles and preferred sleep management strategies (see Table 3).

**TABLE 3:** Nondisabled siblings' accounts on common sleep issues of disabled/ill brother or sister, their roles and preferred sleep management strategies.

Theme	Description	Sample of survey answers
Common sleep issues of disabled or ill brothers or sisters as described by reporting sibling	Settling difficulties Sleep anxiety Sleep very late at night Needing someone to be with him throughout the sleep onset process Sleepless nights Takes a long time to sleep at night	‘Needs to sleep with someone every night and needs his comfort toy’ ‘Struggles to sleep, not asleep till 5/6 am’ ‘Tosses and turns around in bed, throws pillow on the floor’ ‘Sibling sleeps late and ret of family makes sure that DS has everything he needs before’ ‘the last fam member goes to bed, DS ca put himself to bed’ ‘Sibling makes lots of noise prior to sleeping’ ‘Yes, difficult to fall and stay asleep due to ADHD and, constantly wakes up during the night, which wakes neurotypical sibling too’
	Night‐waking Needing the loo Making lots of noise Restlessness	‘Rarely sleeps through the night, wakes up in the middle of the night after 4–5 hours of asleep and then wakes up early, while awake during the night, someone needs to be with him’ ‘Acute insomnia, take medication, runs out of meds, so does not sleep and can be quite agitated and makes noise, keeps everyone else awake or disturbs their sleep, has a tendency to wake up as soon as it is light’ ‘Frequently awake during the night, can have entire sleepless nights that keep mum awake, likely to have seizures at night that require documenting, become severely unwell which may require calling an ambulance or chest percussions to clear secretions’ ‘Needs the loo midnight and needs help getting back to bed’ ‘Wakes up v early and does not go back to sleep’ ‘Yes, DS wakes up for few hours during the night and refuses to sleep’
	Parasomnias Panic attack Breathing problems Difficulty staying asleep Breathing problems Nightmares Eating‐related sleep disorders Nightmares and night terror	‘DS gets up and stands over us’ ‘DS has trouble sleeping, wakes up early and has nightmares’ ‘Has been a difficult sleeper, crying and screaming as soon as DS sibling goes to bed, neurotypical sibling awoken by screams’ ‘Yes, DS has severe sleep apnoea, causing loud snoring.’
	Life‐threatening medical emergencies Sudden unexplained death Seizures Choking	‘Require support with movement, someone needs to check on DS 3–5 times a night to make sure she is not has not had seizure and chokes in her sleep or be injured by it’ ‘Does not sleep throughout the night, increased risk of sudden unexplained death meaning he is vulnerable at night so have 2 monitors that goes off when he seizes’ ‘Every now and then sleep gets disturbed due to sister's seizure’
Roles and responsibilities played by reporting siblings for their disabled or ill siblings	‘Parents first, but I'm always on standby’	‘Parents will check that he has remembered to switch off household appliance and lock doors’ ‘Sibling co‐sleeps and cannot self‐soothe, mum has to stay in bed until deep sleep, can wake up multiple times needs settling down if have night terrors’ ‘Only needs attention when she wakes and screams, parents will go and check, or through the baby monitor’ ‘Parents provide care when sibling seizes’
	Remaining watchful over disabled sibling's needs and safety	‘Pay constant close attention to disabled sibling during the night’ ‘Taking disabled sibling to the toilet’ ‘Assisting parents in night‐time care’ ‘Providing food and drinks during the night for disabled sibling’ ‘Staying wary to provide physical safety against sibling's violent behaviour’ ‘Setting up alarms to check on disabled sibling’ ‘Checking in on disabled sibling to see if they need anything in the middle of the night’ ‘Yes, look out for him during the night as DS will be disoriented when he wakes up’
Strategies reported to support the disabled sibling	Reassurance and settling disabled siblings back to sleep	‘Direct disabled sibling back to bed’ ‘Wakes up a lot and neurotypical sibling helps to calm her’ ‘Adhering to personalised sleep routines, a mix of what works for him’ ‘Sibling needs really soft blanket to sleep, and it take between 20 min and 2 hours to fall asleep’ ‘Sibling will do own things, take meds, study/chill until get tired’ ‘A mixture of personal activities but always setting aside time for providing care for their disabled sibling.’ ‘Tidy house, play sports at the common, make supper, eat supper, tidy up, get brother to bed, tidy own room, have bath, do own work, watch TV, read, go to bed’ ‘Talk to friends, cook, yoga, read or watch tv, sometimes I work on a puzzle or knit, I cook, I call my brother and talk to him until he gets bored or annoyed, I go on social media’

##### 3.1.1.1 Activities Reported From 4 pm up to Bedtime

All participant siblings mentioned that they engage in a variety of activities such as preparing for work for the next day or completing outstanding daytime tasks. If they do not feel pressure to comply with daily work demands, they report confidence to engage with activities that help them to ‘switch off’ and unwind their mind. All siblings mentioned some activity on social media, spending time with friends and household chores.

The most common bedtime was between 10 p.m. and 11 p.m., and the most common wake time was between 7 a.m. and 8 a.m. though a considerable number of siblings wake up between 5 a.m. and 6 a.m. The average number of hours asleep was between 8 and 9 h of sleep, which is within the recommended number of hours of sleep by National Sleep Foundation (Hirshkowitz et al. 2015). For participants who experienced night wakening, most of them fell back to sleep within 10 to 30 min.

For 19 participants, caring responsibilities comprised a significant part of their time before and during bedtime. These include picking up their neurodivergent sibling from school, arranging supportive activities for neurodivergent siblings (movie, comfort food), overseeing medication and putting their neurodivergent siblings to bed. Younger participant siblings (8–12 years old) were observed to have more time to engage in personal activities, whereas older participant siblings (13–25 years old) assumed more caring responsibilities. Parental support, when available, was reported to play a positive role in ensuring that participant siblings had space and time to engage in their own personal activities. This support meant that they had less pressure before bedtime.

Participant siblings were keenly aware of any specific routines that their neurodivergent sibling required, such as putting on or turning off specific environmental stimuli or allowing engagement with specific topics/materials. Such personalised sleep routines were reportedly helpful in settling neurodivergent siblings for bedtime and varied widely from family to family.

Neurotypical siblings were aware that any situations causing distress to their neurodivergent sibling may lead to greater settling difficulties and later sleep times for everyone in the family.

##### 3.1.1.2 Common Sleep Struggles

The majority of the participants (85%) reported that their siblings experience difficulties with falling asleep or staying asleep. Fewer mentioned symptoms or diagnosis of sleep apnoea and nightwalking.

Neurotypical siblings reported that they are ‘watchful’ and ‘ready to support or redirect’, and in all cases, they reported that their priority is the safety and comfort of their siblings. None of them mentioned receiving professional support regarding sleep issues.

#### 3.1.2 Neurotypical Siblings Reporting on Their Own Sleep

Table 4 presents a sample of codes and descriptions in relation to how siblings report on their sleep habits, their support needs and day‐functioning disturbances.

**TABLE 4:** Nondisabled siblings reporting on their own sleep.

Theme	Description	Sample of survey answers
Main reasons for sleep disturbances for reporting siblings	Night‐time caring responsibilities *N* = 25	‘Sibling having a meltdown’ ‘Need to take him to toilet, night terrors’ ‘He had a bad day and anxiety is high’
	Disabled sibling's specific sleep habits or sleep problems *N* = 30	‘Vocal tics’ ‘Sleep apnoea’ ‘Meltdowns’ ‘Laughter’ ‘Screaming’ ‘Walking around the house’
	Personal anxieties *N* = 35	‘Racing thoughts, dwelling on past issues, worry about the family’ ‘Anxieties, Down Syndrome (DS) loud vocal tics, DS walking around the house’ ‘Anxiety, more so because live away from family’ ‘Anxiety, worrying about the future’ ‘Anxiety, not really’ ‘Worry about DS sibling and mum’
Effects of sleep disturbances on daytime functioning of reporting siblings	Physical effects and weariness	‘Very tired and struggle to concentrate toward the end of the day’ ‘Tired during the day and finding it hard to get up in the morning, hard to concentrate doing work’ ‘Feel very tired and do not want to get up in the morning, cannot concentrate’ ‘Very tired the next day at school’ ‘Dependence on caffeine’ ‘Hard to stay awake on way to school’
	Poor cognitive performance	‘Makes it hard to concentrate, hard to perform to best of ability’ ‘Very tired and struggle to concentrate toward the end of the day’ ‘Half asleep in the morning during morning lectures’ ‘Frequently tired and drink a lot of coffee to focus, focus does not last long because so tired’ ‘Hard to stay awake and concentrate, constantly tired and sleep pattern is incompatible with work schedule’
	Disrupted social‐psychological functioning^a^	‘Feel more tired during the day at work, causes worry as being a nurse, sleepiness leads to poor functioning at work like making mistakes’ ‘Get grumpy because tired’ ‘When need to work, tiredness can increase anxiety’ ‘Become irritable, struggle to concentrate’ ‘Get tired at random times, feel bad when cannot help parents’
	Does not affect daytime functioning	‘Often tired but not really to do with DS’ ‘None’ ‘Does not affect me that much’
Support for sleep disturbances for reporting siblings	Informal sources as first line of help for nonanxiety‐related sleep disturbances	‘Nothing, mum tells everyone to go to bed’ ‘Good friends’ ‘Nothing at the moment, it is being investigated, managed at home by mum’ ‘Do not get any advice and do not want to tell friends because don want to worry parents about it’ ‘Do yoga and mindfulness exercises, partner used to help her get back to sleep due to anxieties but being single now, self soothe or talk to housemate if cannot sleep, neurotypical sibling has long episodes of insomnia’ ‘Talk to mother and some close friends in school, but no formal help’
	Types of formal help sought and the experiences of it	‘Circadian medicine’ ‘Have had sleep tests in hospital, prescribed sleep medication, seen doctors’ ‘See a psychotherapist once a week’ ‘Told by doctors that since sleep issues were related to disability, I was not entitled to any help, in school, suggested me to move in with a relative but wasn't doable’
	Dealing with anxiety	‘Gone to Improving Access to Psychological Therapies (IAPT also known as Talking Therapies) counselling for mental health concerns’ ‘Good friends’
	Environmental changes to facilitate better sleep	‘Parents put on the fan if sibling is shouting’ ‘Have to sleep in mum's room if keep getting disturbed’ ‘Go to mum and sleep with her, dogs will help neurotypical sibling know if someone comes into her room’ ‘Mum says if they win the lottery they will move to England as Ireland has very little support’ ‘Trying to get mum to separate rooms but no space’
	Not getting any help (either because do not experience disturbances, no access to help or did not seek help)	‘None’ ‘None, this is an ongoing issue that never gets resolved’ ‘Does not need help but occasionally bed wets’ ‘Nothing’ ‘None’

^a^ We define socio‐psychological functioning as one's ability to effectively engage and cope with social and psychological aspects of life such as taking care of themselves or engaging in social activities.

##### 3.1.2.1 A Personalised Team Effort to Manage Sleep Struggles of Disabled Sibling

All participant siblings were aware of the needs of their disabled siblings during the night. Most neurotypical siblings reported settling difficulties as a common problem for their disabled sibling, who often takes a few hours to fall asleep. Most disabled siblings do not adhere to a prescribed set of sleep routines by health experts, suggesting that families have developed for themselves routines that best suit the needs of their disabled child.

##### 3.1.2.2 Main Reasons for Sleep Disturbances for Participant Siblings

About 50% of the neurotypical siblings were not providing care that would impact their sleep. Only 20% of participant siblings reported that their sleep is often disrupted by their disabled sibling's night‐time behaviours; this included ‘disabled siblings often wake up due to nightmares’, ‘eating‐related sleep disorders’ and ‘enuresis and night stirrings’—including shouting, screaming and walking around the house. Such events are reported to cause greatest sleep disturbance amongst participant siblings compared with any other cause. A smaller number of participant siblings must remain awake throughout the night because of medical emergencies or to ensure that sleeping positions are appropriate particularly when seizures and breathing difficulties were reported. Parents taking turns in sleepless nights was the only reported support for the siblings.

Five siblings reported that although they did not have any physical involvement at night‐time with their disabled siblings, their anxieties about their disabled sibling, family and futures were the main cause of a bad night of sleep.

##### 3.1.2.3 Daytime Effects of Sleep Disturbances on Neurotypical Siblings

Tiredness, poor concentration and reliance on stimulants were the most reported effects of poor sleep on daytime function. School‐aged neurotypical siblings faced behavioural difficulties, moodiness and poor concentration in school, which has reportedly led to them ‘getting into trouble’. Neurotypical siblings who needed to work faced performance problems at work which threatened to ‘jeopardise their careers’ and relationship with superiors, some even reporting that sleepiness and fatigue has led them to ‘call in sick’ the next day. Because of fatigue, moodiness and physical discomforts from the lack of sleep, neurotypical siblings experience a decline in daytime function, often reportedly leading to feelings of exhaustion and frustration.

##### 3.1.2.4 Support Needs for Sleep Disturbances

Although many neurotypical siblings relied on short‐term solutions for treating daytime sleepiness, less than 20% of them sought formal help for their sleep struggles that related to struggles falling asleep or staying asleep. The siblings perceived as the key issues related to their struggle to fall asleep their ‘anxiety about things will go during the night’ or ‘anxiety thinking about the future’. All siblings valued formal and nonformal support for common worries by speaking with family members, seeing a psychotherapist and engaging in relaxation activities. Challenging behaviours of their disabled sibling in the night were managed by parents and physical environmental adjustments to facilitate good sleep were undertaken by parents or neurotypical siblings themselves. Where formal help was sought, circadian‐rhythm medication, counselling and sleep tests were common solutions undertaken. Neurotypical siblings reported a mix of effectiveness for the help they received from professionals, with some reporting that they have been dismissed because their sleep problems were not directly related to them but their disabled sibling.

#### 3.1.3 Siblings' Experiences of Loneliness and Support Needs

The nondisabled siblings' experiences of loneliness, support needs and daytime functioning are presented in Table 5.

**TABLE 5:** Nondisabled siblings' experiences of loneliness.

Themes	Theme description	Evidence from data
Perceived lack of understanding and acceptance from wider society	Wider society does not understand what it is like living with disabled sibling	‘People who do not understand’ ‘No one really understands what it is really like’ ‘Peers in school/uni. not understanding’ ‘No one understands the situation’ ‘Nobody understanding how it is like to be them’
	Standing out and being stigmatised because of perceived differences	‘Being different to everyone else around because of disabled sister’ ‘Spending childhood playing alone’ ‘Not having a life like their friends’
Carer's lonely journey	Assuming a heavy and lonesome responsibility	‘Wondering: Who cares for the carer? Who listens to the listener?’ ‘Thinking about the future when neurotypical sibling needs to care for disabled sibling without anyone to help’ ‘Having no other siblings to share the responsibility with’ ‘Increased level of responsibility’ ‘Worrying about the future by oneself’ ‘Caring for sibling alone in the future’ ‘Not having others share similar experiences’
	Fear of over‐burdening parents	‘Not wanting to share with parents as do not want to burden them’ ‘Not being able to speak out to parents about it’ ‘Do not want to worry parents’
	Misplaced attention: Unrecognised accomplishments and competing for parent's attention	‘Accomplishments not perceived to be important’ ‘Parents being busy with sibling’ ‘Attention being given to someone else all the time’ ‘Having less attention’ ‘Nobody bothering with them’
	Lack of opportunities for leisure and socialisation	‘Not being able to meet others outside the home due to care’ ‘Friends cannot come round often’ ‘Not being able to go to restaurants’
Growing up faster than peers	‘No time to be silly’	‘Having to be much more mature than peers’ ‘Do not have the knowledge to be silly’
	Keeping watchful over the family from a young age	‘Not engaging in social activities like drinking as need to be alert to react to emergency at home’
	Having different interests and priorities as peers	‘Distancing from peers’ ‘Difficult to learn how to socialise’
Unspoken relational struggles and types of support	Invalidations of own feelings and envy towards other people's lives	‘Nobody in the same situation so not worth sharing experiences’ ‘Deep rooted idea that one's feeling cannot compare’
	Lack of meaningful connections and company	‘Not feeling connections with people’ ‘Do not like being alone’ ‘Having no one to speak to’ ‘Empty feeling’ ‘Not being able to connect with others’ ‘Isolated and alone regardless of physical or social situation’ ‘Not having anyone to be with’ ‘Not having connections’ ‘Not being able to share deep thoughts with’
	Reluctance to reach out	‘Finding it difficult to talk about it at the moment’ ‘Suffering in silence, not being able to talk about issues’
	Psycho‐somatic symptoms	‘Empty feeling’ ‘Knot in the stomach’ ‘Missing something’

##### 3.1.3.1 Perceived Lack of Understanding and Acceptance From Wider Society

Loneliness was defined by participant siblings as a general sense of lack of understanding and empathy from the wider society. Neurotypical siblings face experiences that their peers may not have encountered. Because of their caring roles, they report being attuned to the needs of their neurodivergent sibling, taking on more caring responsibilities and having less time for leisure activities and socialisation. They may also engage in activities from a very young age that their peers may find strange like changing catheters, tube‐feeding, clearing mucus, fixing splints and managing the challenging behaviours of their disabled sibling in public. Siblings reported that during periods of intense medical emergencies for the neurodivergent child, per neurotypical siblings have found the need to hide, lie or diminish the seriousness of the condition as they do not want peers to be frightened, judgemental or to unfriend them.

Noteworthy are the shift in definitions of loneliness as neurotypical siblings grew older. Child neurotypical siblings defined loneliness as a lack of friends, teenage neurotypical siblings defined loneliness as being judged by their peers and young adult neurotypical siblings defined loneliness as a lack of awareness in the wider society.

Loneliness can mean so many things but having nobody to relate or understand your perspective can be horrifically lonely.

I've always found that my friends understand the impact having a neurodivergent brother has on the larger aspects (anxiety etc) but don't consider the impact on everyday things, like planning time. (25‐year‐old sister from England living with 24‐year‐old brother with profound and multiple learning disabilities caused by a hypoxic brain injury and multiple strokes because of meningococcal septicaemia)

Peers who are unable to relate to the experiences of young carers may engage in stigmatisation and mockery. However, neurotypical siblings' identities are closely related to their disabled siblings, and the need to dissociate or put on a false or socially acceptable identity has led to feelings of isolation and loneliness. Having different lifestyles to their peers, many of them do not conform to adopting normative hobbies and interests commonly associated with similar‐aged peers, leading to feelings of being judged for being their authentic self.

Loneliness is a big part of my life. I feel lonely because no one really understands what it is like, and my parents are often busy with my siblings. I can't talk to my friends about it because they don't understand what it is like, and they don't really know how much of a big deal it is. (13‐year‐old sister from England living with 16‐year‐old autistic sibling).

##### 3.1.3.2 Carer's Lonely Journey

Understanding their roles as sibling young carers and the toll it has on their parents, most neurotypical siblings have an implicit assumption that they should not share their concerns or worries with their parents or friends because they ‘do not want to overburden them’. However, neurotypical siblings reported experiencing depression and anxiety and having no one to speak about this with. Neurotypical siblings also tend to invalidate their strong feelings by thinking that their ‘feelings cannot compare’ to their parents or even their disabled siblings. Neurotypical siblings also reported a lack of leisure time and few opportunities to go out as a family to restaurants and shopping centres, even though these would have helped neurotypical siblings to relax and bond with their families. As neurotypical siblings get older, they realise the weight of the responsibility and the possibility that they would one day be the sole care provider for their disabled siblings. This “weight of responsibility and future carer role” could seem like a daunting and lonely prospect especially for neurotypical siblings who are still in their childhood and adolescent years.

In terms of having a neurodivergent sibling I often feel lonely as I have no other siblings to share the responsibility with. It can be quite isolating also to not have friends who understand that I will one day be responsible for my brother. (22‐year‐old sister from England living with 11‐year‐old brother with cerebral palsy)

Misplaced attention as a form of loneliness meant that neurotypical siblings perceived less attention being given to them because ‘parents' attention was always going to disabled siblings’ and ‘parents were always busy with disabled siblings’. Parents spent more time with disabled siblings because of medical appointments, caring requirements and fulfilment of daily activities. This “divided attention from parents” has led to a perceived need for neurotypical siblings to compete for parent's time and attention. Neurotypical siblings also felt that their achievements were unrecognised because, since their disabled sibling found everyday tasks extremely challenging, parents often overlook and rarely publicly acknowledge neurotypical siblings' significant achievements.

I struggle with feeling lonely as I'm the only sibling that has a role in my brother's care and feel that I have a responsibility to help my parents but do not want to burden them with how I feel on top of what they already deal with. I also feel like my issues/accomplishments are not important as my brother's life is so complex. (20‐year‐old sister from Wales living with a 12‐year‐old brother with Dravet syndrome—complex epilepsy, autism, ataxia, development delay)

Neurotypical siblings also shared those common experiences for their peers, like birthday parties and going out with friends, are limited because of a lack of leisure time and a possibility that something embarrassing or inconvenient might happen when friends come over to their house.

Lonely when my friends cannot come around as often as I'd like as my brother has a constant meltdown when we cannot go to restaurants and certain places as my brother will get cross. (13‐year‐old brother from Northern Ireland living with 9‐year‐old nonverbal autistic brother with severe learning disability, challenging behaviour, obsessive compulsive disorder [OCD] and attention deficit hyperactivity disorder [ADHD])

##### 3.1.3.3 Growing Faster Than Peers

Many neurotypical siblings felt that they must grow up faster than their peers. Neurotypical siblings often must keep alert, understand the specificities of their disabled sibling's medical needs and avail themselves to help family members assume caring roles under short notice. Because these were performed from a young age, young neurotypical siblings may not differentiate between their lives and those of their peers, but this difference comes clearer into view as neurotypical siblings approach their teenage years. As they gain more independence and develop curiosity to explore the outside world, neurotypical siblings reported that they may not have time for socialisation or the know‐how to ‘be silly’. As a result of this, neurotypical siblings who are also sibling young carers feel reluctant to join in activities with their peer groups. Neurotypical siblings also reported having very different life priorities to their peers, often involving thinking about the future and being able to support their families as their life's priorities.

At school, when I was younger, I was so much more mature than my peers. I had changed nappies and rung ambulances and been left in charge. Distance from me and my peers made it difficult to learn to socialise … I do not have some of that knowledge of how to be silly, how to behave in a group of my peers. (25‐year‐old sister from England living with 16‐year‐old brother with Angelman's syndrome, epilepsy, autism, severe learning difficulty, dyspraxia, nonverbal)

##### 3.1.3.4 Unspoken Relational Struggles and Types of Support

Informal sources of help for loneliness were sought more than formal ones. Where formal help was sought, psychological therapies such as counselling, mindfulness and meditation provided the mental health support required. However, neurotypical siblings provided feedback that help sought from general practitioners were met with dismissal as well as further referrals to organisations, who were themselves lacking in sufficient funding to provide any efficacious help. Engaging in hobbies and belonging to close‐knit community groups provided the safe space neurotypical siblings needed to socialise and reach out, particularly through networking with fellow neurotypical siblings. Sibling support groups have received praise for their important active roles in the lives of neurotypical siblings including being a safe space to share difficult struggles, exchanging information and normalising the difficult experiences neurotypical siblings encounter daily. They are also praised for providing ample training opportunities for not only neurotypical siblings but the community at large, including schools, parents and the public, who wish to understand more about the lives of neurotypical siblings of a disabled and/or chronically ill sibling as well as how to support them.

When I was younger, I would have really appreciated feeling seen and understood ‐ seeing and speaking with other siblings would have helped me understand my feelings of being ignored, as well as loneliness and guilt. Understanding that other people go through the same things as you is so important and is often lost on younger children. Simply sharing our rather unique experiences and insights can do so much to help. (18‐year‐old sister from England living with a 21‐year‐old sister with cerebral palsy)

Siblings highlighted how schools can play a vital role in helping them succeed educationally such as eliminating exclusion measures for poor externalising behaviour, increasing access to psychological therapies within schools and lesson‐based aides for *neurotypical* siblings to catch up on lessons should they require to take time off to provide care.

## 4 Discussion

In this study, we aimed to provide a deeper insight into the experiences and interplay of loneliness, sleep experiences and needs of siblings of neurodivergent brothers and sisters. To our knowledge, this is the first study to focus on neurotypical siblings' experiences of loneliness and sleep while caring for a neurodivergent sibling.

Qualitative findings highlighted the prominent role of loneliness experienced by siblings without disabilities. The participants offered various reasons such as demands for caring responsibilities that are rarely understood by others and limited personal time, which can potentially limit opportunities for socialisation and peer relationships. Similarly, lonely individuals tend to be less satisfied with their friendships and typically have fewer close friends (Locke et al. 2010), and they also experience peer rejections at higher rates (Achterbergh et al. 2020; Wang et al. 2020). A common theme that was discussed evidently was siblings' feeling of being neglected and their hesitation to seek help which aligns with previous work by Fleitas (2000) showing that siblings may often feel isolated and left out. Loneliness was also articulated as sentiments of stigma and being solely responsible not only to support sibling but also parents and not to be a burden to anyone around them. Siblings often experienced feelings of loneliness due to a perceived lack of understanding and acceptance by the wider society and due to the carer's lonely journey as they usually felt that they had no one to talk to about their struggles. Several siblings also felt they were growing faster because of their increased responsibilities, and as a result, it was difficult for them to engage in fun activities with their peers according to their age. Siblings also reflected on their struggles with different types of external formal support. Their specific needs as siblings of disabled people were often dismissed by professionals who had limited understanding of how to accommodate them, and at the same time, services were often underfunded to provide tailored support and resources.

Sleep disturbances were found to be less because of disruptions by the disabled sibling's night‐time sleep behaviours and more because of worries and anxieties about their disabled sibling, family and futures. Participant siblings either under‐reported issues in order not to create worry to others around their needs or, when raising concerns around their sleep, such concerns did not seem to be perceived worthy of legitimate focus of clinical attention. It is also possible that the personalised strategies that were implemented by the reporting siblings in our study to support their disabled or ill sibling ensured that they obtained sufficient sleep. Most siblings were able to speak about sleep with a family member or engage in relaxation activities. Parents often play protective roles in promoting optimal sleep for their disabled and nondisabled siblings (Pavlopoulou 2022; Wright et al. 2011). Finally, our data indicate to an extent that loneliness may be a greater hindrance to daytime functioning than sleep disturbances as Hawkley and Cacioppo (2010) noted. Future research could further elucidate whether different factors such as loneliness and sleep problems may contribute differently to various aspects of siblings' daytime functioning and use actigraphy and sleep diaries as well as interviews. Further research should also shed light into formal and informal support beyond including personalised versus mainstream sleep routines and their effectiveness for all family members. Given that sleep issues are more common amongst children with physical disabilities, which constitutes a minority (less than 5%) of the sample in this study, extra emphasis should be placed on this group.

### 4.1 Strengths

Overall, the present study has several strengths that contribute to its overall robustness and validity. Firstly, building upon a participatory framework, we involved adult siblings, sleep trainers and charity leaders with extensive experiential and professional expertise in sibling relationships to help us refine research questions, methods, results and dissemination plans. This has probably enabled us to capture more genuinely the nuances in siblings' accounts. Secondly, the study included a population of individuals with diverse chronic illness/disabilities and conditions, which enhances the generalisability of our findings to a broader population. Finally, its online format allows for wider access and participation from individuals who may have difficulty attending traditional research settings because of other commitments or health conditions also feeds into the study's strengths. Online formats of research often equate to inclusivity and ensure that the usually unheard voices and experiences of marginalised people are represented and valued equally. This inclusivity extended to our survey participants, whose ages ranged from 8 to 25 years old. For younger neurotypical siblings, the survey was completed with the help of their parents, ensuring accurate and thoughtful responses. This is of particular importance for this group given their caring responsibilities. This study elicited participants from convenient samples, from a large and heterogeneous sibling population. None of the participant siblings reported being neurodivergent themselves and relied on self‐reported data, which may not always be accurate due to factors like social desirability bias.

### 4.2 Implications to Practice

It is not uncommon for siblings' experiences and struggles to be invalidated and dismissed by healthcare professionals who have limited awareness and understanding of how to support them. Our findings have the potential to inform current policy and clinical practice including training provision to educate professionals about the barriers related to sleep difficulties experienced by siblings of young people with disabilities or chronic illness. Similarly, our conclusions highlight the importance of supportive networks as safe, trusted spaces in which siblings can connect with other siblings and build friendships, share common strengths and struggles, engage in entertaining activities and exchange more targeted information and resources. It is important to understand and normalise the issues faced by siblings, encourage peer support and raise awareness amongst parents and carers about the impact of disabled sibling's sleep on all their children. Parental support and clear personalised night‐time routines for the disabled sibling could ensure that neurotypical siblings have more personal time to focus on their self‐care, for example, by engaging in their preferred self‐soothing activities. Training for healthcare visitors and sleep councillors should emphasise the need for parents to be mindful of the sleep of all family members with a focus of siblings' experiences, perspectives and needs. Such professionals should keep siblings updated with what is happening in the family system and also consult them for any support plans and consider their expertise and experiences. Similarly, our findings could be of high practical value for schools and school‐focused mental health services as they underline the central role that family‐level factors (e.g., home routines) and other social factors such as peer relationships (e.g., lack of understanding/acceptance and stigmatisation) play in young people's well‐being. Considering the wider focus on building whole school approaches that promote a culture of respect (Public Health England 2021), it is necessary to create safe school spaces in which young siblings feel they are part of accepting and welcoming school communities, and there are assigned people to listen an engage with their worries or offer proactively opportunities to be involved in extra curricula activities and hobbies (Kassa and Pavlopoulou 2021). Parents may find it useful to share with sibling's teacher aspects of their day‐to‐day caring responsibilities and give them ideas to acknowledge the role of the student as a sibling and to celebrate the positive aspects of being a sibling of a disabled child.

### 4.3 Conclusions and Future Directions

Our research on the experiences of loneliness and sleep habits of siblings of brothers/sisters who are chronically ill or disabled has provided some key insights. However, there are limitations to the studies conducted thus far that need to be addressed in future research. To begin with, there is a limited representation of accounts from young people across different genders and ethnicities and their intersections. This means that the experiences of certain groups may not be fully captured in research findings and evidence shows that certain groups experience higher instances of mental distress and loneliness, such as LGBTQIA+ groups or neurodivergent siblings with caring responsibilities of their brothers and sisters. Further to this, it is important to stress that the qualitative data are derived from a survey and not interviews, thus providing robust but not in‐depth narratives. Finally, our survey lacked data on the age caring responsibilities started on various activities (e.g., changing catheters and tube‐feeding). Hence, future research should aim to explore further this significant area through interviews and focus groups to gain a more nuanced understanding of siblings' lived experiences. Future research should also build on these findings to explore the intersections between these issues and other factors such as race, sexuality and socio‐economic status to ensure that these findings are more generalisable and thus more likely to have practical implications for siblings with different backgrounds and needs. Further to this, researchers also should examine the association between the age caring responsibilities start and mental health. Finally, more in‐depth qualitative interviews will shine more light on understanding the source of sleep difficulties for the siblings.

## Author Contributions

**G. Pavlopoulou:** conceptualization, data curation, writing – original draft, formal analysis, supervision, methodology, writing – review and editing, project administration, visualization. **E. Sim:** conceptualization, data curation, writing – original draft, writing – review and editing, methodology, formal analysis. **S. Peter:** formal analysis, writing – original draft, writing – review and editing, visualization. **Gardani, M:** writing – original draft, writing – review and editing, formal analysis. **V. Beevers:** conceptualization, writing – original draft, writing – review and editing, methodology. **C. Kassa:** conceptualization, writing – original draft, writing – review and editing, methodology. **V. Sideropoulos:** formal analysis, visualization, writing – original draft, writing – review and editing, project administration, methodology.

## Ethics Statement

The study obtained ethical approval from the Dissertation Ethics Committee in the Department of Psychology & Human Development at IOE, UCL's Faculty of Education and Society. All respondents provided online consent to participate in the study.

## Supporting information


**Table S1** List of author‐created survey questions.

## Data Availability

The data that support the findings of this study are available on request from the corresponding author. The data are not publicly available due to privacy or ethical restrictions.
