# Supplementary material for: ‘Who Listens to the Listener, Who Cares for the Carer?’ A Cross‐Sectional Study of Social Connectedness and Sleep Experiences of Young Siblings of Neurodivergent People
Source: Child Care Health Dev. 2024 Dec 4;51(1):e70014. doi: 10.1111/cch.70014 (PMC11616255; doi:10.1111/cch.70014)
Supplement: Supplementary file 1 — Table S1 List of author‐created survey questions. [file CCH-51-e70014-s001.docx]

**6. Supplementary Materials**

Supplementary Table 1. List of author-created survey questions.

| **Topic Area** | **Examples of Qualitative Questions** |
| --- | --- |
| General Information | - Please state ways in which a sibling support group might help you… - What are the kinds of help/support that your sibling needs (e.g., on a daily basis)? |
| Sleep | - This is what I do from teatime (4pm) till bedtime: - Does your sibling have issues that impact you/other family members? - Are you providing care or assistance to your disabled and/or chronically ill brother or sister (or perhaps helping a parent who is supporting them) in the night? - Do the special conditions in which your disabled and/or chronically ill siblings place their equipment for their medical condition, like the position of their wheelchair, breathing and respiratory equipment, feeding equipment etc, or any other medical reasons, affect your sleep? Please describe how it affects you. - If you do experience sleep disturbances, what are the main reasons for them? (e.g., anxiety, being worried about your sibling's condition). - What kinds of help do you get for the sleep disturbances that you experience? They can be from professionals (like doctors, clinics, school, mental health services, community support groups) or from people whom you know (like parent/guardian, close friend, extended family, teacher etc). Any Other comments on sleep issues? - How does your sleep affect you in the daytime at school/work? |
| Loneliness | - What does loneliness mean to you? - When do you feel lonely and what do you do when you feel lonely? - How does having a disabled sibling affect your perspective about friendships/relationships? - What kinds of help do you get for the experiences of loneliness that you might have? |
| Support Needs | - How do you think schools can help you as you provide care for your disabled sibling? - What advice would you give to fellow siblings like yourself? (on the issue of sleep, loneliness, day-time function) - What advice would you give to fellow siblings like yourself? (on the issue of sleep, loneliness, day-time function) - How are you caring for your brother/sister differently because of Covid? And how has this affected your experiences of sleep and loneliness? |
| Aspirations | - What are your aspirations, hopes and dreams for the future for your sibling? - What are your aspirations, hopes and dreams for the future for your yourself? - 3 things I hope people around me will understand about having a sibling with a disability and/or chronic illness: - If you could be granted 3 wishes, what would they be? / 2. What would your ideal day be like? |
